# Synthesis and Absorption Properties of Hollow-spherical Dy_2_Cu_2_O_5_ via a Coordination Compound Method with [DyCu(3,4-pdc)_2_(OAc)(H_2_O)_2_]•10.5H_2_O Precursor

**DOI:** 10.1038/s41598-017-13544-4

**Published:** 2017-10-12

**Authors:** Xuanwen Liu, Junhua You, Renchao Wang, Zhiyuan Ni, Fei Han, Lei Jin, Zhiqi Ye, Zhao Fang, Rui Guo

**Affiliations:** 1 School of Resources and Materials, Northeastern University at Qinhuangdao, Qinhuangdao, 066004 China; 2 School of Materials Science and Engineering, Northeastern University, Shenyang, 110004 China; 3 Key Laboratory of Nano-Materials and Photoelectric Catalysis of Qinhuangdao, Qinhuangdao, 066004 China; 4 School of Materials Science and Engineering, Shenyang University of Technology, Shenyang, 110870 China

## Abstract

Dy_2_Cu_2_O_5_ nanoparticles with perovskite structures were synthesized via a simple solution method (SSM) and a coordination compound method (CCM) using [DyCu(3,4-pdc)_2_(OAc)(H_2_O)_2_]•10.5H_2_O (pdc = 3,4-pyridinedicarboxylic acid) as precursor. The as-prepared samples were structurally characterized by X-ray diffraction (XRD), scanning electron microscopy (SEM), high-resolution transmission electron microscopy (HRTEM), x-ray photoelectron spectroscopy (XPS) and standard Brunauer–Emmett–Teller (BET) methods. Compared to the aggregated hexahedral particles prepared by SSM, the Dy_2_Cu_2_O_5_ of CCM showed hollow spherical morphology composed of nanoparticles with average diameters of 100–150 nm and a larger special surface area up to 36.5 m^2^/g. The maximum adsorption capacity (*Q*_*m*_) of CCM for malachite green (MG) determined by the adsorption isotherms with different adsorbent dosages of 0.03–0.07 g, reached 5.54 g/g at room temperature. The thermodynamic parameters of adsorption process were estimated by the fittings of the isotherms at 298, 318, and 338 K, and the kinetic parameters were obtained from the time-dependent adsorption isotherms. The results revealed that the adsorption process followed a pseudo-second-order reaction. Finally, the adsorption mechanism was studied using a competitive ion (CI) experiments, and the highly efficient selective adsorption was achieved due to strong O-Cu and O-Dy coordination bonds between Dy_2_Cu_2_O_5_ and MG.

## Introduction

Over the past few decades, hazardous waste remediation has emerged as an alarming national and international concern^1–11^. Dye-containing wastewaters discharged by industries are particularly problematic pollutants because dyes, such as methyl orange (MO), methylene blue (MB), rhodamine B (RhB), malachite green (MG) and so on, are too stable to be biodegraded in human body^12^. For instance, MG is an organic dye and drug that could enter the human food chains through fish and water, causing human poisoning. Therefore, the developments of dedicated and effective adsorbents or photocatalysts for dyes are very important^13–15^. To date, a number of physical, chemical and biological methods have been developed and tested with variable advantages and drawbacks. Among these processes, the adsorption technology gained a rapid growing due to its low cost.

One particular challenge of adsorption research is to design selective adsorbents, which could not only preserve the useful organic matter in solutions, but also may be used for by-product separation during organic synthesis. Another challenge is how to increase the special surface area of the adsorbents and increase the maximum adsorption capacity. Based on the strong O-Cu and O-Dy bonds and inspired by the work of Y. Li *et al*.^4^, we prepared Dy_2_Cu_2_O_5_ and observed strong selective adsorption for MG. Therefore, the corresponding kinetic and thermodynamic experiments were carried out in details.

The preparation of nano-/micro-materials with novel and controllable morphologies became an important research topic due to fundamental and application of adsorption materials^16–18^. In this aspect, the nanocrystallization of ZnO and ZrO improved their adsorption performance for MG^19–21^. Another kind of strong adsorbents was mesoporous materials (i.e., ordered mesoporous carbons and mesoporous poly(acrylic acid)/SiO_2_)^22,23^. Among them, flowerlike ZnO was reported with the largest *Q*_*m*_ (maximum adsorption capacity) of 2587 mg/g. But few adsorbents were reported with selective properties for MG. Therefore, the studies of the selective adsorption properties of Dy_2_Cu_2_O_5_ for MG are interesting.

CCM is considered as a promising strategy for designing and synthesizing materials with controllable shapes, sizes, and dimensions^24–30^. In this study, hollow-spherical Dy_2_Cu_2_O_5_ samples were prepared by CCM to yield particles with large special surface areas when compared to those prepared by SSM. The as-prepared Dy_2_Cu_2_O_5_ particles were characterized by using X-ray diffraction (XRD), scanning electron microscopy (SEM), high-resolution transmission electron microscopy (HRTEM), x-ray photoelectron spectroscopy (XPS) and standard Brunauer–Emmett–Teller (BET) methods. Compared to catalysts and adsorbents based on transition metal or rare earth oxides, only a few reports are available on properties of Ln_2_Cu_2_O_5_-type rare earth cuprates. To the best of our knowledge, the present report is the first to discuss selective adsorption activity of A_2_B_2_O_5_-type rare earth cuprate towards MG with significant *Q*_*m*_ values reaching up 5.54 g/g at room temperature.

## Experimental

### Synthesis

The reagents used in this study were all of analytical grade and were used without further treatment. Typically, the SSM samples were prepared according to the literature^29,30^. Under constant stirring, Cu(OAc)_2_·4H_2_O and Dy(NO_3_)_3_·5H_2_O were dissolved in distilled water at the stoichiometric proportions. After 1 h, the solution was heated to a gel and then calcined at 900 °C for 1 h to yield Dy_2_Cu_2_O_5_.

The CCM precursor [DyCu(3,4-pdc)_2_(OAc)(H_2_O)_2_]•10.5H_2_O was prepared according to procedures published in the literature^29,30^. Cu(OAc)_2_·4H_2_O, Dy(NO_3_)_3_·5H_2_O, 3,4-pdc, and triethylamine with corresponding stoichiometric proportions were dissolved in a mixture of water-methanol at the volume ratio of 1:1. The solution was stirred for 3 h then filtered off and allowed to stand until the formation of blue single crystals. Blue block crystals suitable for X-ray analysis were obtained by filtration, washing with ethyl ether and drying in air. The obtained single-crystal precursor was then calcined at 900 °C for 1 h under N_2_ atmosphere, followed by 0.5 h in air to yield Dy_2_Cu_2_O_5_.

### Characterization

The diffraction data of the coordination precursor [DyCu(3,4-pdc)_2_(OAc)(H_2_O)_2_]•10.5H_2_O were collected at 296(2) K by a Rigaku Saturn CCD diffractometer equipped with graphite monochromated *Mo-K*_*α*_ radiation using the *ω*-scan technique. The Data were processed using CrystalClear software and corrected for Lorentz and polarization effects^31^. Absorption corrections were applied using a multiscan program, and the structures were solved by direct methods then refined by full-matrix least squares based on *F* ^2^ using SHELXTL program package^32^.

XRD patterns were recorded on a D/Max-RB X-ray diffractometer (Rigaku) using Cu *Kα* irradiation at a scan rate (2θ) of 0.05°/s from 10 to 90°. Powder morphologies were monitored by SEM (Zeiss Supra 55) and HRTEM (FEI Tecnai F30), in addition with the capability of taking energy dispersive X-ray (EDX) spectra. The specific surface areas of the as-prepared samples were measured by N_2_ adsorption/desorption experiments at 77 K with a Builder SSA-4300 instrument. The XPS measurements were performed on a PHI 5000 C ESCA System with Mg *K* source operating at 14.0 kV and 25 mA.

### Adsorption experiments

To evaluate the adsorption capacity of Dy_2_Cu_2_O_5_ towards MG, Batch adsorption tests were carried out. The adsorption isotherm experiments were conducted with various adsorbent doses (0.03–0.07 g) in 1000 mL of 0.4 g/L MG aqueous/ethanol mixture solution at 1:1 volume ratio and different temperatures (298, 313, and 338 K) for 7 days. Once equilibrium was established, 5 mL of the suspension was removed and the adsorbent particles were removed by centrifugation. The concentration of MG was determined using UV-Visible (RF 5301) spectrophotometry at 618 nm. The adsorbed amount was calculated according to Eq. 1.

1 $${q}_{e}=\frac{({C}_{0}-{C}_{e})\times V}{m}$$

where *q*_*e*_ (mg/g) is the adsorption capacity at the equilibrium concentration, *C*_0_ (mg/L) and *C*_*e*_ (mg/L) are respectively the initial and equilibrium concentrations of MG in the solution, *V* (L) is the initial solution volume, and *m* (g) is the weight of the used dry adsorbent.

The kinetic characteristics of the adsorption process were analyzed based on the time response of the isothermal adsorption experiments with 0.05 g adsorbent at 298–338 K, as described above but prior to equilibrium. The initial CI concentrations in the competitive ion experiments were fixed to 0.02 mol/L.

## Results and Discussion

### Characterization

**Figure 1:**
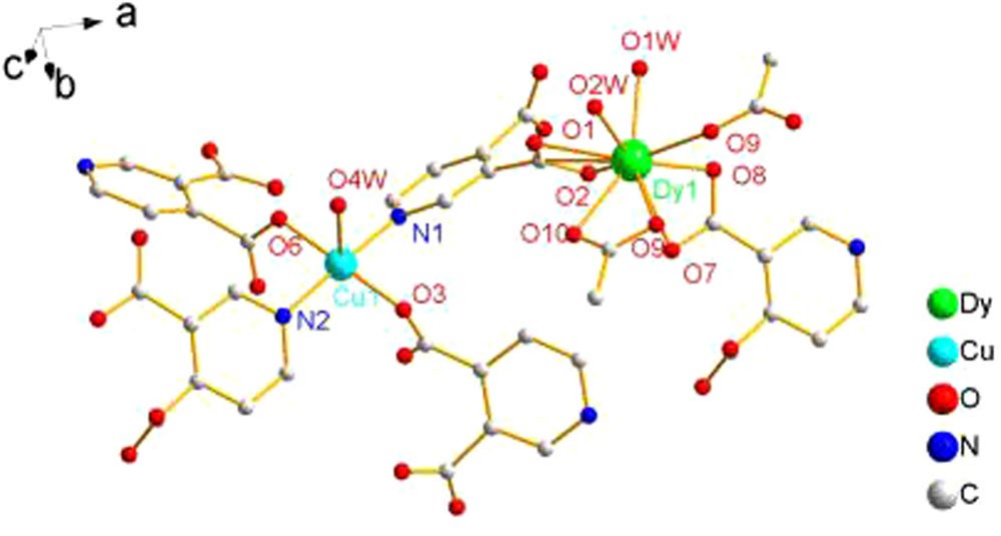
Local coordination environments of Dy^3+^ and Cu^2+^ ions in the coordination precursor (hydrogen atoms and lattice water molecules are omitted for clarity).

**Figure 2:**
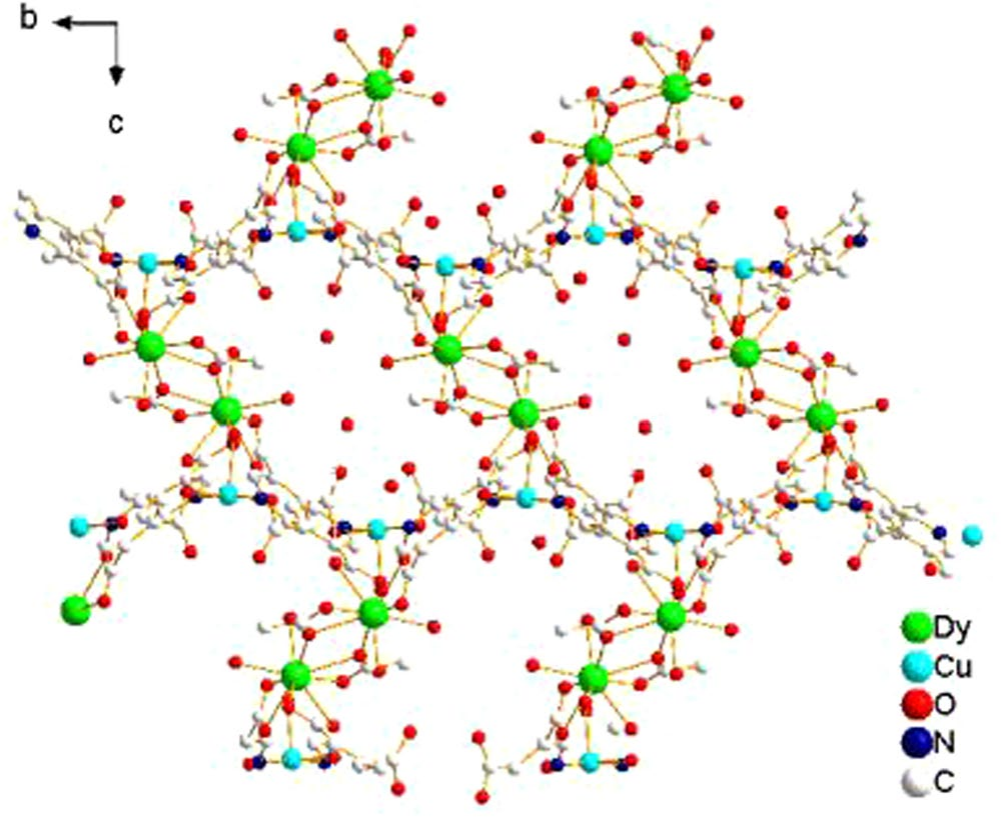
The framework of the coordination complex viewed along the a axis.

The crystal structure of the coordination compound, which is depicted in Figs 1 and 2, consisted of asymmetric [DyCu(3,4-pdc)_2_(OAc)(H_2_O)_2_] units with different orientations. The crystal data and selected bond lengths/angles are shown in Table S1 and S2, respectively. Each repeat unit contained one Dy^3+^ unit and one Cu^2+^ unit. Dy^3+^ is in a distorted tricapped trigonal prismatic coordination environment with a DyO_9_ core: four coordinated oxygen atoms are derived from two 3,4-pdc ligands, two oxygen atoms of coordinated water molecules, and three coordinated oxygen atoms from two acetate ligands. The Cu^2+^ is five-coordinated with tetragonal-pyramidal geometry, where the equatorial plane is occupied by two N (N1, N2) and two O (O3, O6) atoms from four different 3,4-pdc ligands, clearly following the trans-effect. The axial position is occupied by one water molecule. Because of the Jahn-Teller effect, the axial Cu1–O5 distance (0.2237 nm) is larger than the other Cu–O distances (0.1980(5) and 0.1981(5) nm). Both the 3,4-pdc ligands adopt a quadridentate chelating-bridging mode to chelate one Dy^3+^ ion and link two Cu^2+^ ions. The acetic acid ligand connects two Dy^3+^ ions using two O atoms to chelating-coordinate with one Dy^3+^, and one of the two O atoms bridge the other adjacent Dy^3+^ ion. Cu^2+^ ion, which is bridged by four 3,4-pdc ligands, act as a 4-connected node, resulting in a wavelike (4,4)-connected 2D planes (002). These 2D planes are packed along the *c* axis and further linked by dinuclear Dy^3+^ units, resulting in the 3D coordination framework shown in Fig. 2.

**Figure 3:**
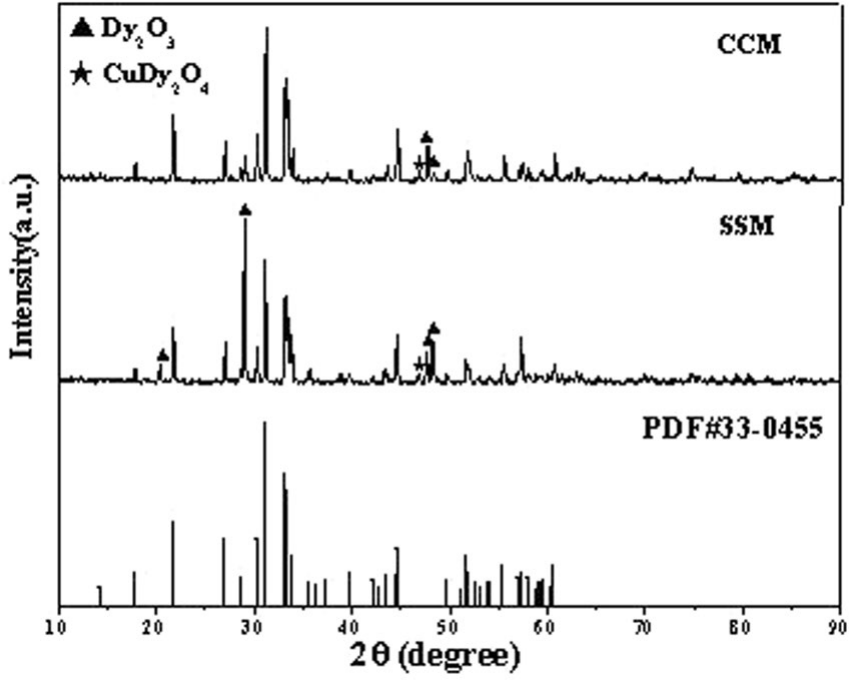
XRD patterns of Dy_2_Cu_2_O_5_ prepared through SSM and CCM in comparison with PDF# 33-0455.

XRD was used to investigate phase structures of the as-prepared Dy_2_Cu_2_O_5_ particles, and the results are shown in Fig. 3 in comparison with PDF# 33–0455. It can clearly be seen that most of the diffraction peaks agreed well with PDF# 33-0455 and the few impurity peaks were assigned to Dy_2_O_3_ and CuDy_2_O_4_. Compared to SSM, the sample of CCM was purer, which could be conjectured by the fact that the metal ions were evenly distributed in the coordination precursor. When organic molecules were thermally decomposed, a mobile phase was provided to help the ions migrate better and forms small orientated single crystal.

**Figure 4:**
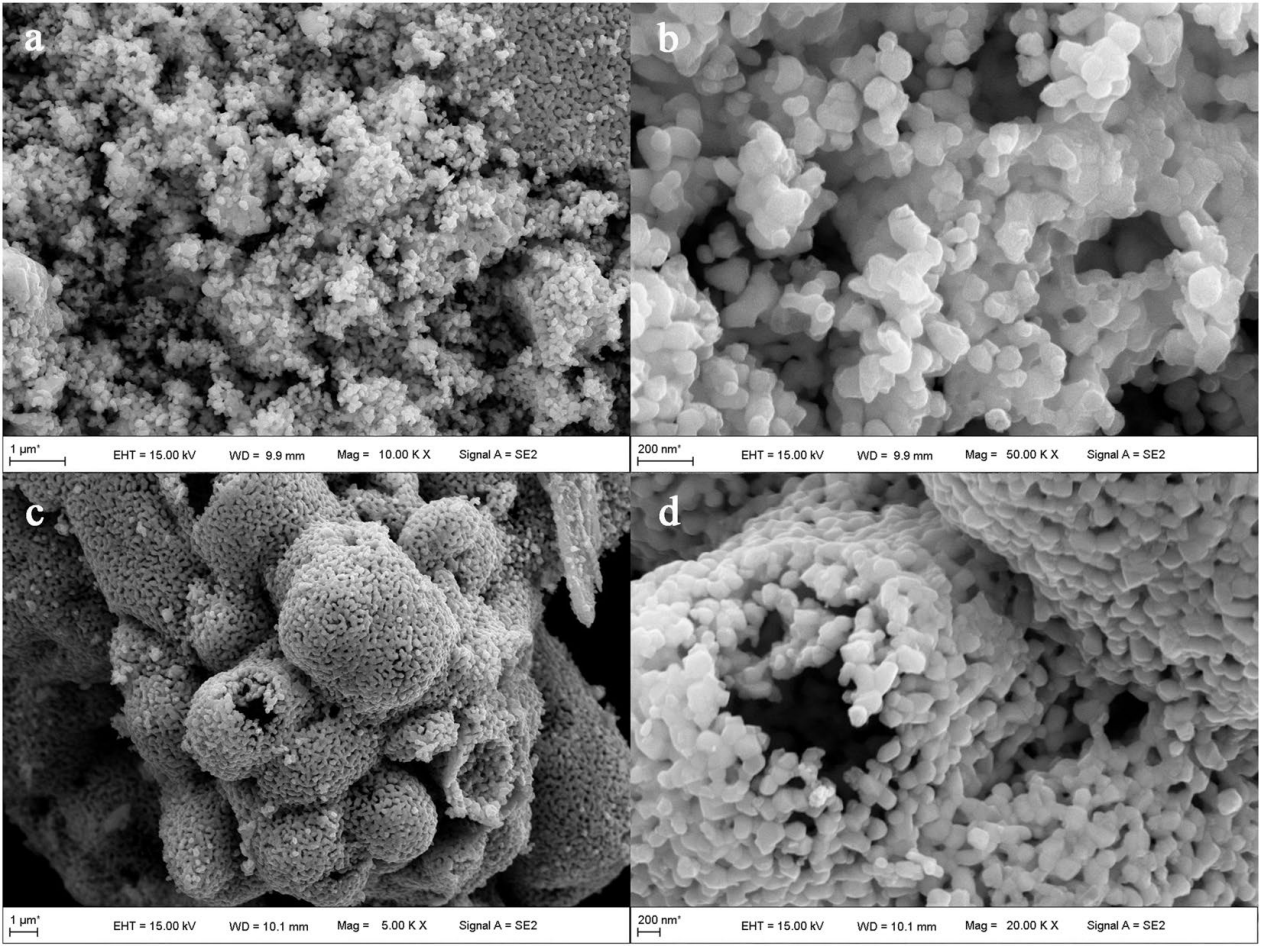
SEM images of Dy_2_Cu_2_O_5_ of SSM (**a,b**) and CCM (**c,d**) at different magnification.

Figure 4 illustrates the SEM images of Dy_2_Cu_2_O_5_ prepared by SSM and CCM. A few sand-like aggregates with grain sizes ranging from 100–150 nm were observed for the SSM samples (Fig. 4a and b). The small grains looked hexagonal prismatic in nature, and the particles below the surface were sintered together (Fig. 4b). The sample contained a large number of pores with uneven pore sizes, large enough for dye molecules and water molecules to penetrate and pass through.

The low-magnification SEM of the sample prepared by CCM revealed hollow Dy_2_Cu_2_O_5_ microspheres with diameters ranging from 5–10 um with tendencies to aggregation (Fig. 4c). The spherical shell consisted of 1–3 layers of 100–150 nm small particles with large numbers of holes (Fig. 4d). This would allow a facile penetration of organic molecules to reach inside the spheres. These apparent morphologies suggested that thermal decomposition of organic molecules during the calcination process expanded the coordination complex and formed bubbles. As the temperature rose, metal ions present in the bubble walls gradually crystallized to form Dy_2_Cu_2_O_5_ hollow spheres.

**Figure 5:**
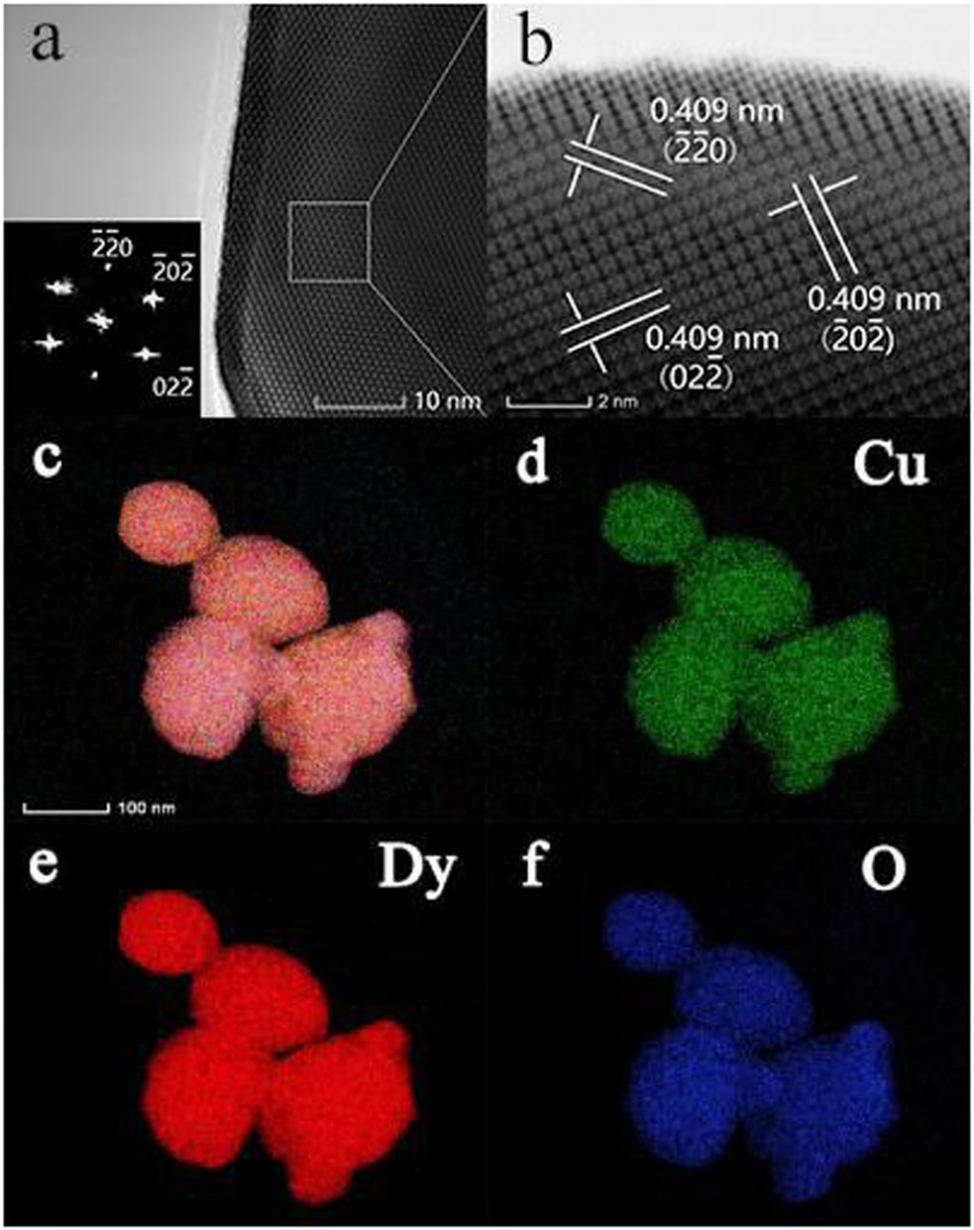
HRTEM images of Dy_2_Cu_2_O_5_ (**a,b**) and elemental mapping of total elements (**c**), Cu (**d**), Dy (**e**) and O (**f**).

To determine the composition of the nanostructures of Dy_2_Cu_2_O_5_ prepared by CCM, further analysis using HRTEM, SAED and EDX elemental mapping of Cu, Dy and O were performed and the data are gathered in Fig. 5. The crystal structure of bulk Dy_2_Cu_2_O_5_ was difficult to analyze by HRTEM because of the high thickness of the sample but some regions at the edges could be processed. The clear lattice spacings detected by HRTEM suggested that Dy_2_Cu_2_O_5_ particles were highly crystalline in nature with single crystalline structure.

The SAED patterns contained three sets of diffraction peaks indexed to (−2–20), (−20–2), and (02–2). These peaks were assigned to 3 sets of lattice plane spacings of about 0.409 nm with different orientations. Therefore, the particles were enclosed possibly by {220}. The elemental mappings of Cu, Dy and O indicated uniform distributions of these elements (Fig. 5d–f), consistent with the XRD data (Fig. 3).

**Figure 6:**
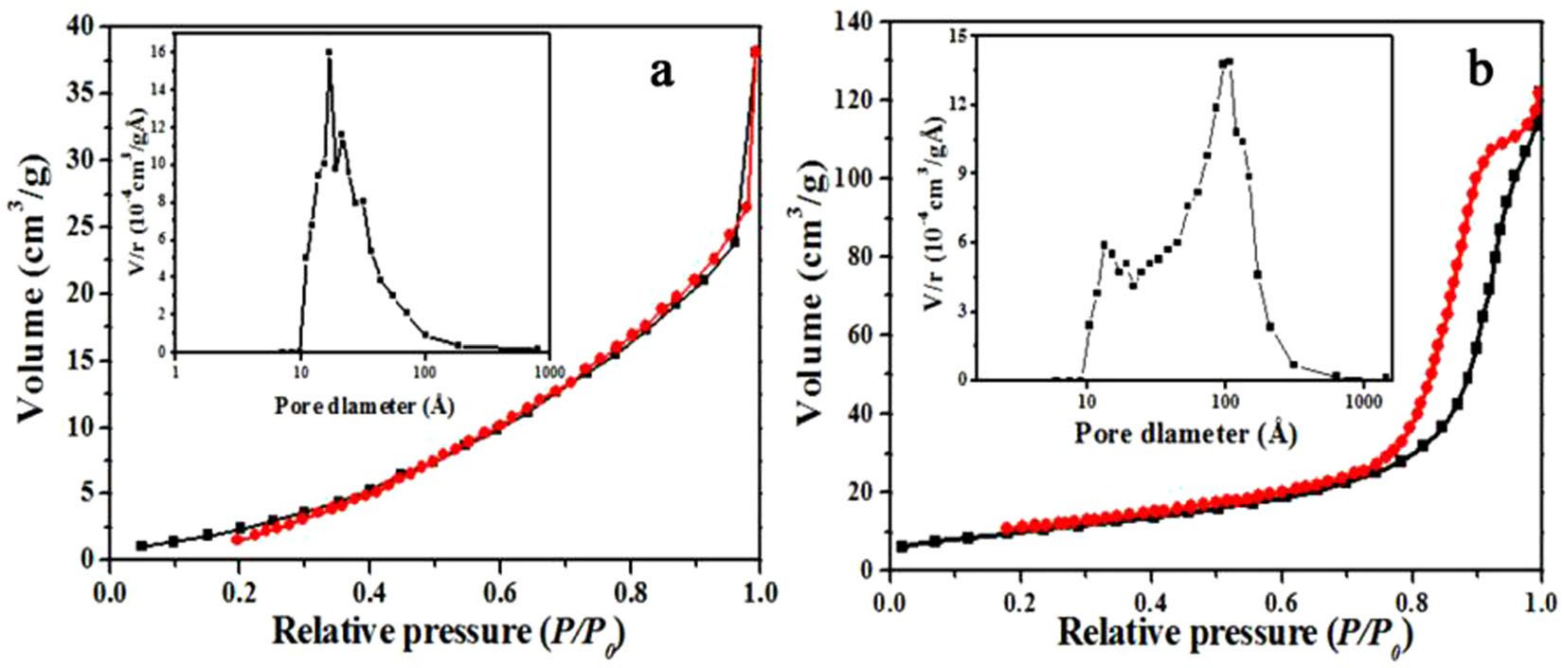
Nitrogen adsorption–desorption isotherms and corresponding pore size distributions of Dy_2_Cu_2_O_5_ of SSM (**a**) and CCM (**b**).

Figure 6 illustrates the nitrogen adsorption-desorption isotherms and pore size distributions of Dy_2_Cu_2_O_5_ prepared by SSM and CCM. The Dy_2_Cu_2_O_5_ of SSM showed type III isotherm profiles according to IUPAC classification, indicating weak adsorbent-adsorbate interaction without the appearance of “ink-bottle”pores between particles^33,34^. By comparison, the Dy_2_Cu_2_O_5_ of CCM depicted type III isotherm curves with approximate H2 hysteresis loop. These adsorption-desorption isotherms were attributed to the presence of pores with narrow necks and wide bodies^33^. These descriptions fit more with hollow spherical shell morphology, consistent with the SEM images shown in Fig. 4.

Moreover, the specific surface area increased from 15.6 m^2^/g for SSM to 36.5 m^2^/g for CCM products, confirming the advantages of the CCM method. The pore size of CCM samples could be divided into two groups: pore sizes ranging from 10–30 nm assigned to the gaps between particles and 30–300 nm assigned to hollow spheres.

**Figure 7:**
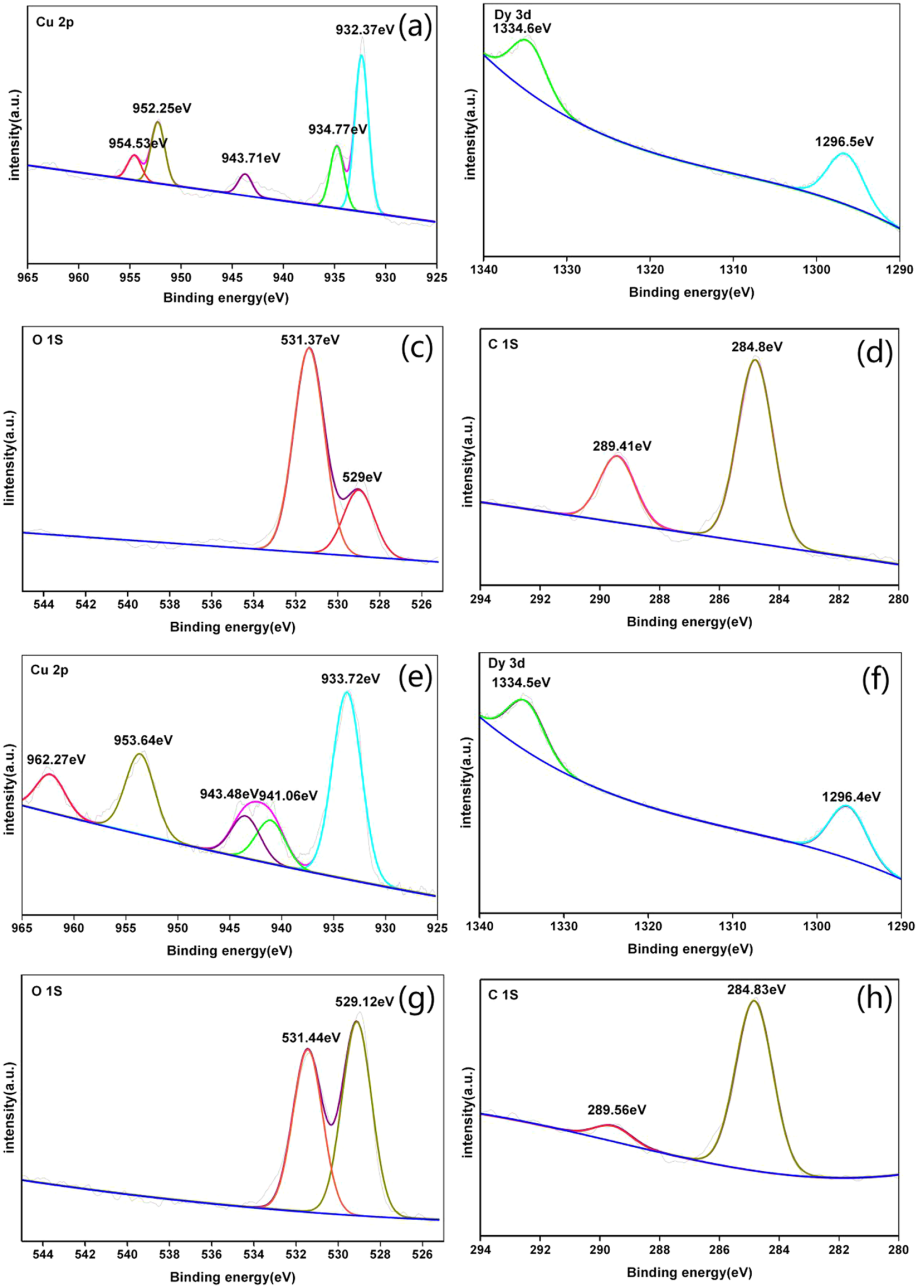
High-resolution XPS spectra of Cu, Dy, O, and C in Dy_2_Cu_2_O_5_: (**a–d**) for CCM and (**e–h**) for SSM.

The valent states of Cu, Dy, O, and C in Dy_2_Cu_2_O_5_ samples prepared by CCM and SSM were characterized by XPS and the results are gathered in Fig. 7. The high-resolution XPS spectra of Cu, Dy, and O were attentively deconvoluted considering spin-orbit coupling. As shown in Fig. 7a, the Cu 2p XPS of CCM showed core level of Cu 2p spectral region with two spin-orbit doublets. The main peaks presented Cu 2p_1/2_ at 952.25 eV and Cu 2p_3/2_ at 932.37 eV with an energy difference of about 20 eV, which could be attributed to Cu ion in CuO_4_ group with formal charge of +2^35^. The second doublet with binding energies at 954.53 eV and 934.77 eV could be ascribed to emission from Cu 2p_1/2_ and Cu 2p_3/2_ core levels of Cu atoms with more positive charges, denoted as +2 + δ_1_, suggesting another inequivalent CuO_4_^35,36^. Meanwhile, the medium peak located at 943.71 eV might be attributed to satellite peaks of Cu 2p, possibly caused by Cu ions with lower coordination numbers on edges and corners of the surface. The atomic ratio of Cu with different charges was estimated to 1:1, implying that inequivalent Cu ions could alternately be distributed in the lattice^37^.

The high-resolution XPS spectra of Dy 3d in sample prepared by CCM are shown in Fig. 7b. The peaks of 3d_5/2_ and 3d_3/2_ observed at respectively 1296.7 and 1335.5 eV suggested the presence of chemical equivalent Dy ion with a formal charge of +3^38–40^. Figure 7C showed two different valences of O: at −2 (529 eV) and −2 + δ_2_ (531.37 eV, more positive), indicating the presence of two kinds of non-equivalent O atoms. The binding energies of adventitious carbon (284.8 eV) were applied for charge correction, and the data are summarized in Fig. 7d. The peak at 289.41 eV could be attributed to C-O species^41^. The C residue could be considered as a characteristic of CCM methodology^35^.

By comparison, the XPS profiles of Cu in samples prepared by SSM were more complex (Fig. 7e). The two doublets (933.72, 941.06 eV) of 2p_3/2_ and (953.64, 962.27 eV) of 2p_1/2_ could be assigned to Cu with formal changes of +2 and +2 + δ_3_, respectively. The satellite peak at 943.48 was assigned to surface Cu atom, similar to CCM in Fig. 7a.

Figure 7a and e indicated that Cu in both samples prepared by CCM and SSM contained more positive valence in addition to the standard +2, and low valence proportion of Cu atom was higher in CCM samples. The spectra of Dy in SSM products were greatly consistent with that of CCM samples (Fig. 7f), here the doublets located at 1296.4 and 1334.5 eV corresponded to the +3 charge. The peaks of O in SSM samples (Fig. 7g) were similar to that shown in Fig. 7c, while the proportion of more negative charge (−2 + δ_2_) increased. Hence, C in SSM samples contained less of more-positive-valence C (289.56 eV for C 1 s).

Overall, the CCM samples contained large amounts of carbon residue. This led to the formation of C-O species and induced O ion with more positive valence. Also, the valence state of Cu in CCM compounds was more negative. These findings suggested that the electronic structures were greatly affected by the synthesis method.

### Maximum adsorption capacity of Dy_2_Cu_2_O_5_ for MG

**Figure 8:**
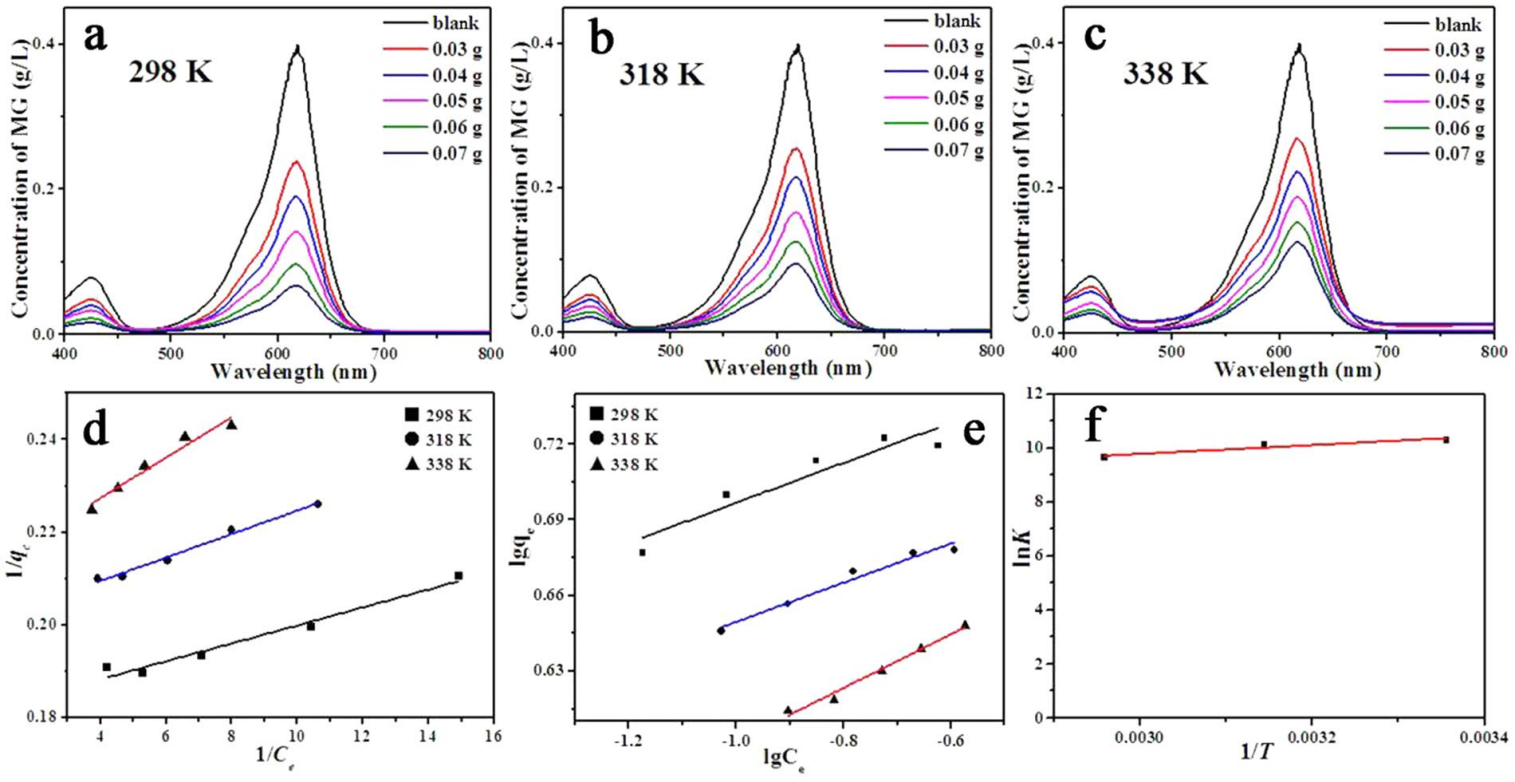
Varieties MG equilibria concentrations in the maximum adsorption experiment of CCM at 298 (**a**), 318 (**b**), and 338 K (**c**) and the corresponding fitting lines according to Eq. (2), Eq. (3), and Eq. (4) are depicted in (**d**,**e** and **f**) respectively.

The adsorption capacities of Dy_2_Cu_2_O_5_ were evaluated using equilibrium adsorption experiments at 298, 318, and 338 K. Figure 8 shows the adsorption isotherms for various absorbent doses of CCM (0.03, 0.04, 0.05, 0.06, and 0.07 g), and the maximum adsorption capacity (*Q*_*m*_) was calculated using the Langmuir model (Eq. 2) and Freundlich model (Eq. 3)^42^.

2 $$\frac{1}{{q}_{e}}=\frac{1}{{K}^{\theta }{Q}_{m}}\times \frac{1}{{C}_{e}}+\frac{1}{{Q}_{m}}$$

3 $$\mathrm{ln}\,{q}_{e}=\,\mathrm{ln}\,{K}_{F}+\frac{1}{n}\,\mathrm{ln}\,{C}_{e}$$

where *K* ^*θ*^ is the Langmuir constants and *K*_*F*_ and *n* are Freundlich constants.

The values of related parameters and *R* ^2^ of CCM samples are listed in Table 1. The *R* ^2^ of Langmuir model was closer to 1 and much larger than that of the Freundlich model, indicating that Langmuir model fitted better the data. The *Q*_*m*_ of the as-synthesized adsorbent estimated by Langmuir model reached 5.54 g/g at 298 K. The maximum adsorption capacities of some selected adsorbents are summarized in Table 2. To the best of our knowledge, the as-prepared Dy_2_Cu_2_O_5_ through CCM delivered the largest adsorption value towards MG. As temperature increased, the maximum adsorption capacity of Dy_2_Cu_2_O_5_ decreased to 4.77 g/g at 338 K. Also, the equilibrium constant (*K* ^*θ*^) decreased from 2.96 × 10^4^ to 1.53 × 10^4^ L/mol as the temperature rose from 298 to 338 K. The thermodynamic parameters were fitted according to Table 1, and the results are shown in Fig. 8 following Eq. (4)^43^:

4 $$\mathrm{ln}\,{K}^{{\rm{\theta }}}=\frac{-{{\rm{\Delta }}}_{r}{{G}_{m}}^{\theta }}{RT}=-\frac{{{\rm{\Delta }}}_{r}{{H}_{m}}^{\theta }}{R}\times \frac{1}{T}+\frac{{{\rm{\Delta }}}_{r}{{S}_{m}}^{\theta }}{R}$$

where *Δ*_*r*_ *G*_*m*_^*θ*^, *Δ*_*r*_ *H*_*m*_^*θ*^, and *Δ*_*r*_ *S*_*m*_^*θ*^ are the standard Gibbs free energy change, standard enthalpy change and standard entropy change for adsorption of 1 mol MG, respectively.

**Table 1:** Partial fitting results obtained from the maximum adsorption capacity experiment of CCM.

*T*(K)	Langmuir			Freundlich		
	*Q*_*m*_ *(g/g)*	*K* ^*θ*^(×10^4^ *L/mol*)	*R* ^2^	*K*_*F*_(*L/mol*)	*1/n*	*R* ^2^
298	5.54	2.96	0.9579	1905	0.0795	0.85718
318	5.02	2.50	0.9829	1702	0.0778	0.95434
338	4.77	1.53	0.9401	1631	0.107	0.97942

**Table 2:** Comparison of adsorption capacities of different adsorbents for MG at 298 K.

Adsorbent	Adsorption capacity (mg/g)	Data resource
Porous C-ZrO_2_ composite	2500	21
Bamboo-based activated carbon	263.58	44
Ordered mesoporous carbons	354.5	22
Poly(acrylic acid)/SiO_2_ membranes	220.49	23
ZnO-activated carbon	322.58	19
Cellulose	458.72	45
ZnO flowerlike architectures	2587.0	20
This work of CCM	5540	

*Δ*_*r*_ *G*_*m*_^*θ*^ was estimated to −25.6 kJ/mol, indicating that the adsorption process was spontaneous. The large absolute value indicated the significance of the adsorption reaction trend. The negative value of *Δ*_*r*_ *H*_*m*_^*θ*^ (−13.6 kJ/mol) suggested that the adsorption process was exothermic, which was consistent with the equilibrium response to temperature. The *Δ*_*r*_ *S*_*m*_^*θ*^ value was recorded as 40.3 J/mol·K, implying that adsorbed MG molecules were greatly disordered on the Dy_2_Cu_2_O_5_ particles, possibly arranged according to different orientations. All these thermodynamic parameters demonstrated that Dy_2_Cu_2_O_5_ particles could be used as efficient adsorbents to remove MG from aqueous solutions. The *Q*_*m*_ values tended to decrease rapidly when the adsorbent dosage was less than 0.04 g at 298 K. The data should follow a linear trend if fitted using corrected Eq. (1) to yield Eq. (5).

5 $${q}_{e}=\frac{({C}_{0}-{C}_{e})\times V}{m-m^{\prime} }$$

A positive value of m′ suggests that there may be a systemic mass loss of adsorbent, while mass loss became negligible when amounts of the adsorbent increased.

By comparison, *Q*_*m*_ of Dy_2_Cu_2_O_5_ prepared by SSM was estimated to 3.87 g/g (Fig. S3), revealing that CCM was more suitable for preparation of absorbents. The *Q*_*m*_ values of samples based on CCM and SSM were not proportional to special surface areas, indicating that the inner surface of the spherical shell in CCM samples adsorbed less MG. This could be due to the huge volume of MG molecules, which restricted their passage through the shells. The latter could also be induced by agglomerated adsorbents able of absorbing greater amounts of MG due to the presence of slits between the particles (Fig. 4b). In fact, dark spots on the container walls were sometimes observed for larger *q*_*e*_ (When the amount of adsorbent is more than 0.07 g). Consequently, ethanol was added to prevent aggregation of the adsorbent particles.

### Kinetic characteristics

**Figure 9:**
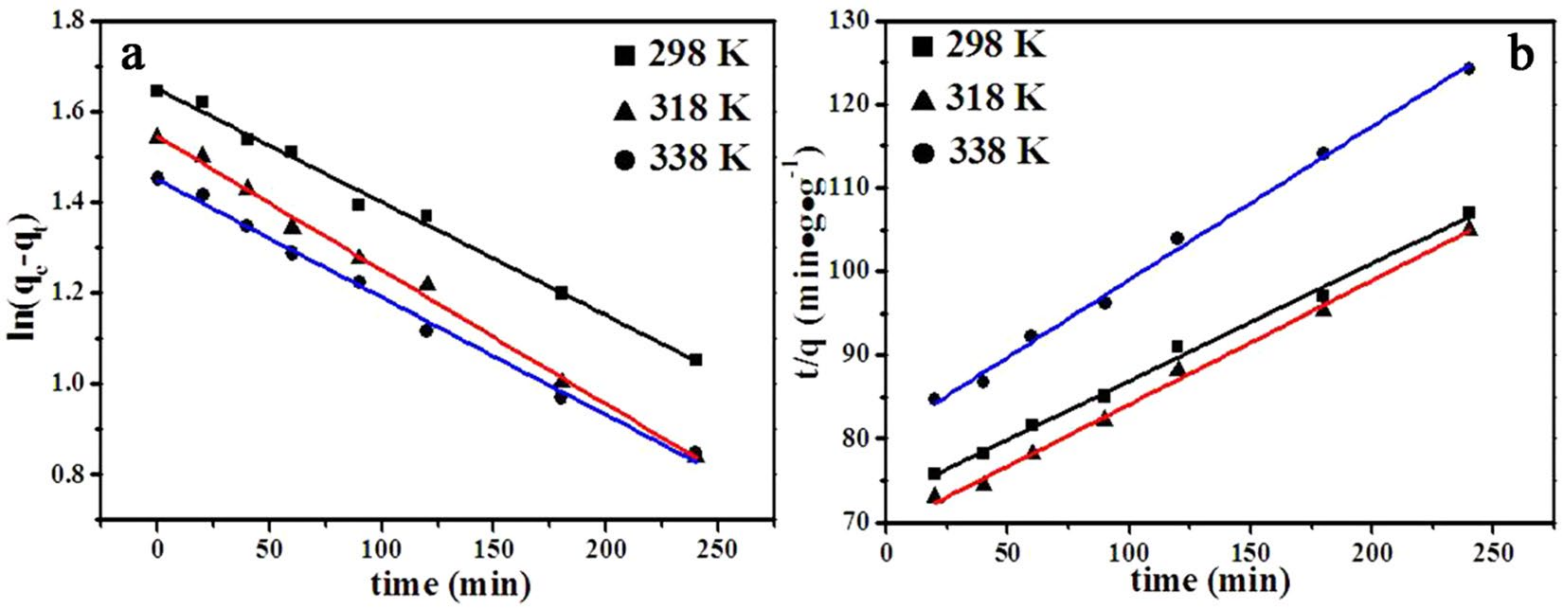
Lines fitted to pseudo-first-order (**a**) and pseudo-second-order (**b**) kinetic models.

The adsorption kinetic studies were performed at 298–338 K, and the adsorption isotherms of 0.05 g Dy_2_Cu_2_O_5_ towards MG are depicted in Fig. 9. The rate of concentration change of MG in the pseudo-first- and pseudo-second-order adsorption processes can be expressed by Eqs (6) and (7).

6 $$\mathrm{ln}({q}_{e}-{q}_{t})=\,\mathrm{ln}({q}_{e})-{k}_{1}t$$

7 $$\frac{t}{{q}_{t}}=\frac{1}{{k}_{2}{{q}_{e}}^{2}}+\frac{1}{{q}_{e}}t$$

where *q*_*e*_ and *q*_*t*_ are the adsorption capacities (g/g) at equilibrium and at time *t*, respectively. *k*_1_ and *k*_2_ are the rate constants of the pseudo-first-order and pseudo-second-order models, respectively.

**Figure 10:**
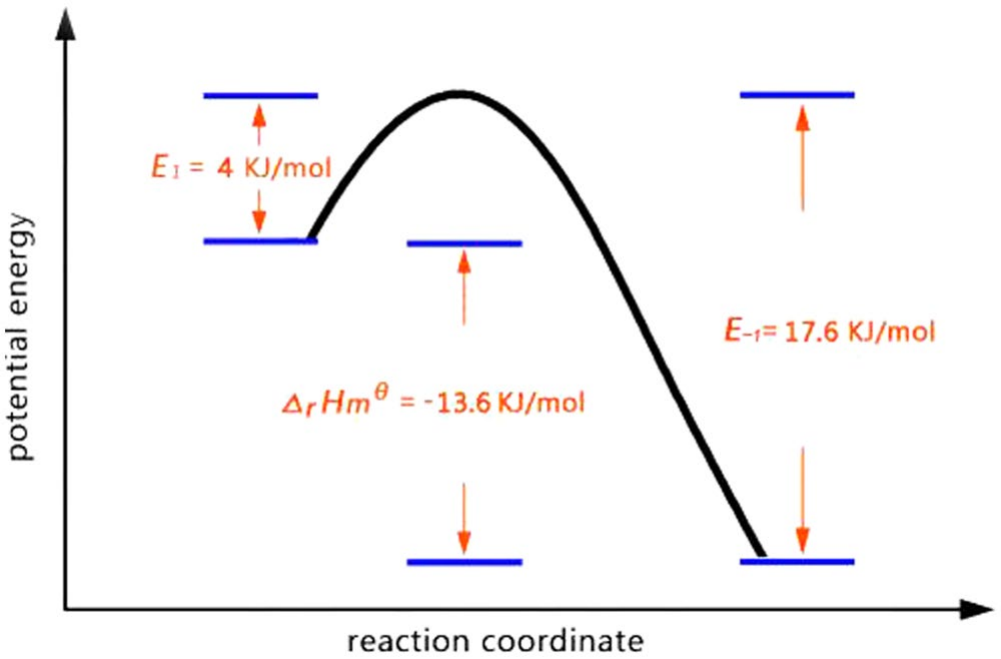
Schematic diagram of potential energy along the reaction coordinate.

As shown in Figs 9 and S4, and Table 3, the changes in concentrations of CCM samples towards MG followed a pseudo-second-order process. Therefore, the kinetic parameters were calculated by fitting the isotherm data in Fig. 9 and Fig. S4 using Eq. (7). Table 3 showed that the *k*_2_ value of CCM samples increased as temperature rose, consistent with predictions from collision theory. The adsorption reaction activation energies (*E*_1_) was fitted according to *k*_2_ values using the Arrhenius formula, and the results are gathered in Fig. 10. The large value of *E*_*−*1_ (the activation energy for desorption process) indicated that the molecular structure of MG greatly changed during the adsorption process. In addition, strong coordination bonds may appear between MG and Dy_2_Cu_2_O_5_ during reaction coordination beyond the transition state.

**Table 3:** Kinetic parameters at temperatures ranging from 298–338 K.

*T*(K)	pseudo-first-order			pseudo-second-order		
	*k*_*1*_(*×10* ^*−*3^ *min* ^*−*1^)	*q*_*e*_(*g/g*)	*R* ^2^	*K*_2_(×10^*−*4^ *g/g·min* ^*−*1^)	*q*_*e*_(*g/g*)	*R* ^2^
298	2.49	5.20	0.9922	2.70	7.13	0.9948
318	2.94	4.69	0.9951	3.16	6.76	0.9954
338	2.59	4.26	0.9944	4.20	5.43	0.9955

### Adsorption mechanism

**Figure 11:**
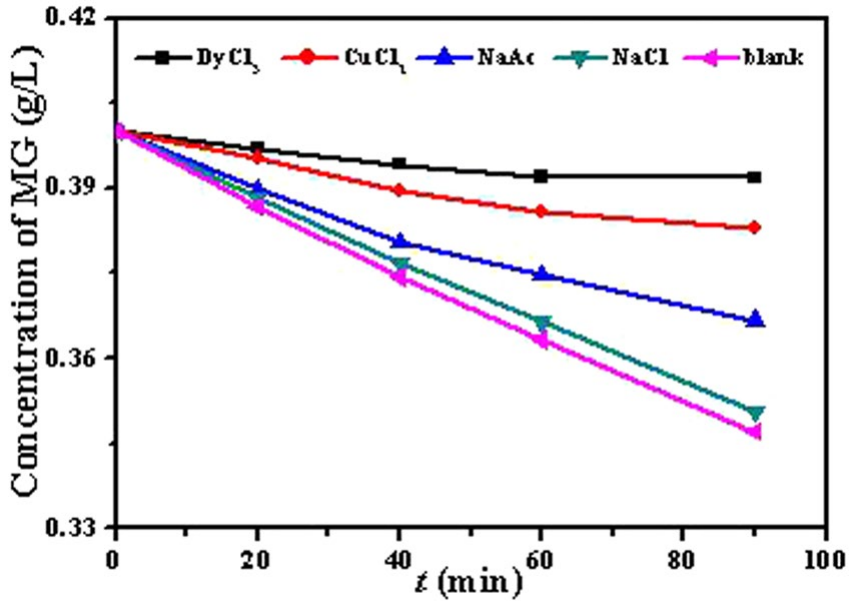
Effects of competitive ions in MG adsorption on Dy_2_Cu_2_O_5_.

To gain a better understanding of the adsorption mechanisms, isothermal adsorption experiments with different competitive ions were carried out and the data are depicted in Fig. 11. The ions (anions like Cl^−^ and cations as Na^+^) showed a little effect on the adsorption process, suggesting that the selective adsorption was different from electrostatic adsorption. The effect OAc^-^ was stronger than that of Cl^-^, due in part to the formation of O-Cu and O-Dy coordination bonds. Also, Cu^2+^ and Dy^3+^ could effectively block the adsorption of MG. These results implied that Cu^2+^ and Dy^3+^ ions were more likely to coordinate with O-containing species, and MG also tended to coordinate with Cu^2+^ and Dy^3+^ in solution.

Overall, these isotherms indicated that the interaction between the adsorbent and adsorbate possibly depended on the O-Cu and O-Dy coordination bonds. Also, the adsorption process might be chemical, where selective adsorption of MG was confirmed by additional experiments showing no adsorption of other dyes like methyl orange (MO) and rhodamine B (RhB)) by the Dy_2_Cu_2_O_5_ adsorbent.

## Conclusions

[DyCu(3,4-pdc)_2_(OAc)(H_2_O)_2_]•10.5H_2_O as coordination precursor was synthesized and structurally characterized by various analytical techniques. Compared to samples prepared by SSM, hollow-spherical Dy_2_Cu_2_O_5_ particles with larger surface areas of 36.5 m^2^/g were successfully prepared by CCM method. The *Q*_*m*_ values of CCM samples increased to 5.54 g/g at 298 K. The adsorption of MG on Dy_2_Cu_2_O_5_ followed a pseudo-second-order exothermic reaction with activation energies *E*_1_ = 4.0 KJ/mol and *E*_*−1*_ = 17.6 KJ/mol. The fitting of the isotherms at different temperatures estimated the thermodynamic parameters *Δ*_*r*_ *G*_*m*_^*θ*^ to −25.6 kJ/mol, *Δ*_*r*_ *H*_*m*_^*θ*^ to −13.6 kJ/mol, and *Δ*_*r*_ *S*_*m*_^*θ*^ to 40.3 J/mol·K. The competitive ion experiments confirmed that the selective adsorption behavior was partially due to O-Cu and O-Dy coordination bonds. Overall, these findings provided new insights into the adsorption properties of selective adsorbents based on Dy_2_Cu_2_O_5_, with potential use in wastewater treatment.
